# Fatty Acid Profile of Cheese from Dairy Goats Fed a Diet Enriched with Castor, Sesame and Faveleira Vegetable Oils

**DOI:** 10.3390/molecules19010992

**Published:** 2014-01-15

**Authors:** Ertha Medeiros, Rita Queiroga, Maria Oliveira, Ariosvaldo Medeiros, Mayara Sabedot, Marco Bomfim, Marta Madruga

**Affiliations:** 1 Graduate Program in Science and Food Technology, Department of Foods Engineering, Technology Center, Federal University of Paraiba (PPGCTA/DEA/CT/UFPB ), Campus I, Joao Pessoa, Paraiba 58.059-900, Brazil; E-Mail: erthajanine@yahoo.com.br; 2 Department of Nutrition, Center for Health Sciences, Federal University of Paraiba (DN/CCS/UFPB), Campus I, Joao Pessoa, Paraiba 58.059-900, Brazil; E-Mail: rcqueiroga@uol.com.br; 3 Department of Nutrition, Federal University of Campina Grande, Cuité, Paraiba 58.175-000, Brazil; E-Mail: elieidynutri@yahoo.com.br; 4 Department of Animal Science, Agricultural Science Center, Federal University of Paraiba (DZ/CCA/UFPB), Campus II, Areia, Paraiba 58.397-000, Brazil; E-Mails: ariosvaldo.medeiros@gmail.com (A.M.); mayara_sabedot@hotmail.com (M.S.); 5 National Center for Goat and Sheep Research, Embrapa, Sobral, Ceara 62.011-970, Brazil; E-Mail: marco.bomfim@embrapa.br

**Keywords:** CLA, diet, goat cheese, nutrition animal, vegetable oil

## Abstract

The addition of vegetable oils to the diets of dairy goats is an alternative to supplemental feeding during the dry period and improves the lipid profile of milk and by-products. Cheeses were produced using milk from cross bred goats (Saanen × Alpina) fed diets enriched with 4% vegetable oil (faveleira, sesame or castor), the fatty acid profile of cheeses was studied. Supplementation with vegetable oils did not increase the total fat percentage of the cheese (*p* ≥ 0.05) but did increase the percentage of CLA isomers, long-chain fatty acids (LCFA) and polyunsaturated fatty acids (PUFA); in addition, the index of desirable fatty acids (DFA - expressed as the sum of unsaturated fatty acids plus stearic acid) was increased for cheese made from milk from goats fed sesame or faveleira oil. Cheeses may have had increased percentages of *cis-*9,*trans-11*-CLA due to the supplementation of animal diets with vegetable oils rich in C18:2, such as faveleira and sesame oils. The fatty acid profile of goat cheese did not change significantly in response to the use of castor oil. Thus, the addition of sesame and faveleira oils to goat diets positively altered the fatty acid profile, which improved the nutritional characteristics of the fat present in goat cheese.

## 1. Introduction

The current focus of the market for animal products has been directed toward the search for foods with lower fat contents. Goat milk is associated with positive health outcomes due to its intrinsic properties, such as low allergenic potential, high digestibility and nutritional value [1]. The lipid composition of goat milk determines its nutritional quality because lipid components, particularly fatty acids, are involved in the production and quality of dairy products and directly affect the taste aspects of milk derivatives [2].

The use of vegetable oils into the diets of goats contributes to the search for alternatives to the dairy goat activity, especially in the dry season (August and January), to increase the nutritional enrichment of dairy products [3]. Among the sources of lipids, faveleira (*Cnidoscolus quercifolius* or *phyllacanthus*), sesame (*Sesamum indicum L.*) and castor (*Ricinus communis L.*) oils have received attention due to their high levels of unsaturated fatty acids [4]; however, the effects of dietary supplementation on small ruminants and the changes in the characteristics of milk and milk products as a result are not well known.

Studies have shown that different milk fat compositions are related to variations in the diets of small ruminants, demonstrating efficiency in increasing the concentration of conjugated linoleic acid (CLA) in milk fat [5,6]. Sanz Sampelayo *et al.* [7] reported that the lipid metabolism in the mammary gland is directly related to milk production and can induce different responses among species of animals (goats and sheep). Other authors have assessed the fatty acid profile of different types of goat cheese of different races [8], maturation times [9], regions [10] and lactation periods [11]. Among them, monounsaturated and polyunsaturated fatty acids, primarily linoleic acid (C18:2), stand out in dairy products (such as cheeses made with goat milk) for presenting concentrations ranging from 1.94% to 3.95% (w/w; Total FAME) [8,12].

Modifications in animal diet can significantly multiply concentrations of bioactive compounds (e.g., CLA and/or omega-3 fatty acids) in milk several times. The most effective strategies involved supplementing ruminant feed with different oils or oilseeds enriched in linoleic or linolenic acids [7,13,14]. However the effects of processing conditions, storage, and aging on the CLA content of various types of cheeses are less relevant. Luna *et al.* [15] employed high temperature conditions in the manufacture of processed cheeses and they found that the production process exerted a negligible effect on the CLA content during manufacture. Although Buccioni *et al.* [9] reported that the total CLA content in Pecorino cheese rose by more than 10% during ripening this increases were lower than when animal diet was supplemented with plant oils [16].

The increase in the CLA content of foods derived from ruminants has attracted attention, for potentially having positive effects on human health due to the anticarcinogenic properties of this fatty acid [17,18], and the intake of the *cis*-9,*trans*-11-CLA isomer in the diet lowers blood cholesterol levels [19]. Given the nutritional benefits of goat milk and its derivatives, the objective of this study was to evaluate the effect of supplementing the diet of dairy goats with faveleira, sesame or castor oils and the influence of this supplementation on the fatty acid profile of cheese.

## 2. Results and Discussion

Supplementation with 4.0% vegetable oil in diets for lactating goats did not promote an increase in the percentage of total fat in goat milk and cheese (*p* ≥ 0.05, Table 1). 

**Table 1:** Means and standard errors of the fatty acid profile (% area ^1^) of cheese made with milk from goats fed with different vegetable oils.

Fatty acids (%)	Treatments (Cheese) ^2^				SEM	Significance
	Control Without oil	“Faveleira” oil	Sesame oil	Castor oil		
**Total Fat **	**23.35**	**24.17**	**22.94**	**22.67**	**0.283**	**ns**
C4:0	0.06	0.00	0.03	0.15	0.027	ns
C6:0	0.91	0.62	0.88	0.74	0.081	ns
C7:0	0.02	0.02	0.02	0.02	0.001	ns
C8:0	2.15	1.88	2.07	2.02	0.048	ns
C9:0	0.06	0.05	0.06	0.06	0.003	ns
C10:0	8.68 ^a^	6.90 ^ab^	6.38 ^b^	8.02 ^ab^	0.315	*
C11:0	0.09	0.08	0.08	0.09	0.004	ns
C12:0	4.44 ^a^	3.08 ^b^	3.28 ^b^	3.73 ^ab^	0.143	*
C13:0	0.03	0.07	0.11	0.09	0.011	ns
C14:0	11.03 ^a^	8.35 ^c^	9.14 ^bc^	10.17 ^ab^	0.268	*
C15:0	0.84 ^ab^	0.67 ^c^	0.72^bc^	0.90 ^a^	0.025	*
C16:0	28.13 ^a^	26.45 ^ab^	24.66 ^b^	27.62 ^ab^	0.485	*
C17:0	0.56	0.42	0.43	0.56	0.016	ns
C18:0	11.88 ^b^	15.13 ^a^	16.64 ^a^	11.63 ^b^	0,607	*
C19:0	0.03	0.05	0.05	0.03	0.003	ns
C20:0	0.31	0.29	0.31	0.32	0.008	ns
C21:0	0.02	0.02	0.04	0.04	0.035	ns
C22:0	0.08	0.05	0.07	0.06	0.005	ns
C23:0	0.02	0.01	0.01	0.04	0.003	ns
C24:0	0.10	0.12	0.14	0.16	0.012	ns
C14:1	0.16 ^a^	0.11 ^b^	0.11 ^b^	0.13 ^ab^	0.007	*
C15:1	0.27 ^a^	0.19 ^b^	0.22 ^ab^	0.29 ^a^	0.011	*
C16:1	0.74 ^ab^	0.80 ^a^	0.68 ^b^	0.83 ^a^	0.017	*
C17:1	0.23 ^ab^	0.18 ^b^	0.18 ^b^	0.29 ^a^	0.012	*
C18:1 n11*cis*	0.49 ^b^	0.83 ^a^	0.78 ^a^	0.30 ^b^	0.050	*
C18:1 n9*cis*	22.70 ^ab^	25.61 ^a^	25.70 ^a^	18.88 ^b^	0.875	*
C18:1 n9*trans*	1.50 ^b^	2.47 ^a^	2.18 ^a^	1.05 ^b^	0.129	*
C18:1 n9*cis*, 12-OH	Nd	nd	nd	6.56	0.346	*
C18:2 n6*cis*	2.77 ^b^	3.48 ^a^	3.35 ^a^	2.57 ^b^	0.095	*
C18:2 n6*trans*	0.05 ^b^	0.06 ^a^	0.06 ^ab^	0.05 ^b^	0.003	*
C18:3 n6	0.11	0.08	0.09	0.11	0.005	ns
C18:3 n3	0.36 ^a^	0.24 ^b^	0.24 ^b^	0.25 ^b^	0.012	*
C20:1 n9	0.03	0.01	0.02	0.02	0.004	ns
C20:2	0.08 ^b^	0.06 ^b^	0.06 ^b^	0.85 ^a^	0.062	*
C20:3 n6	0.05	0.03	0.06	0.04	0.005	ns
C20:4 n6	0.19	0.17	0.19	0.20	0.004	ns
C20:5 n3	0.01	0.00	0.01	0.01	0.001	ns
C22:2	0.26	0.29	0.22	0.29	0.015	ns

^1^ Identified by comparing the retention times of methyl esters of samples with those of standards and were quantified by normalising the areas of methyl esters and expressed as a percentage of area (%); ^2^ 0% oil (control); 4.0% “Faveleira” oil; 4.0% Sesame oil; 4.0% Castor oil. SEM = standard error of the mean. * *p* < 0.05; ns—non significant; nd—non detected; ª Means followed by different lowercase letters in the same row indicate significant differences with Tukey’s test at the 5% significance level.

Thirty-eight fatty acids were identified in the fatty acid profile of goat cheese: 20 saturated fatty acids (SFA), nine monounsaturated fatty acids (MUFA), and nine polyunsaturated fatty acids (PUFA). Among the fatty acids identified, eight constituted 91% of the total areas of the chromatograms: C8:0, C10:0, C12:0, C14:0, C16:0, C18:0, C18:1 and C18:2. Oleic acid (C18:1 n9*cis*) was the fatty acid that contributed most to the profile of unsaturated fatty acids in cheese, and palmitic acid (C16:0) contributed most to the profile of saturated fatty acids.

### 2.1. Fatty Acid Profile

There was a significant difference (*p* < 0.05) in the fatty acids percentage of C10:0, C12:0, C14:0 and C16:0, revealing a downward trend in the percentage of these fatty acids in cheese from goats treated with faveleira and sesame oils in relation to cheese from the control animals. However, cheeses with castor oil had intermediate values for these fatty acids compared to those with the other added oils and to the control with no addition of oil.

The inclusion of vegetable oils in the diet caused a significant increase (*p* < 0.05) in the concentration of stearic acid (C18:0) in cheeses, which increased from 11.88% in the control to an average percentage of 16.64% after treatment with sesame oil and 15.13% with faveleira oil; however, there were no differences in the values (*p* ≥ 0.05) between the castor oil treatment and the control. The increase in the stearic acid concentration can be attributed to extensive ruminal biohydrogenation of the fatty acids C18:3, C18:2 and C18:1 because together, they represent approximately 85 and 67% of the fatty acids of sesame and faveleira oils, respectively [4], wherein the final step of rumen biohydrogenation of linoleic acid is the hydrogenation of acid C18:1 *trans*-11 to C18:0 [20]. This outcome seems to be positive, as part of stearic acid will be transformed into oleic acid by the action of Δ-9 desaturase enzyme in the mammary gland, which promotes the unsaturation of stearic acid (18:0) at carbon 9, producing the oleic acid (C18:1 n9*cis*) present in the final product [21].

The lowest percentage of stearic acid was detected in cheese with castor oil, most likely a result of the strong stability of hydroxyricinoleic acid (C18:1 n9*cis*, 12-OH), present in large amounts in only this oil and hindered the hydrolysis of other fatty acids in the rumen, leading to the formation of hydrogen bonds with the hydroxyl group present in the ricinoleic acid [22]. Consequently, a higher concentration of ricinoleic acid was present in the mammary gland and in the goat cheese, which had percentages of ricinoleic acid greater than 6% and a lower percentage of oleic acid (C18:1 n9*cis*) compared to the other oil treatment groups. Another factor that can be attributed to the reduced amount of stearic acid in cheese made from milk from goats supplemented with castor oil is the low concentration of linoleic acid in castor oil [4].

Another aspect that corroborates the influence of ricinoleic acid in the conversion of vaccenic acid (C18:1 *trans*-11) to stearic (C18:0) is the fact that unsaturated fatty acids can alter the rumen fermentation of fibre by their toxic action on fibrolytic bacteria, which are involved in the biohydrogenation of polyunsaturated fatty acids [23]. Medeiros *et al.* [4] reported that among the three oils studied here, castor oil showed the highest percentage of unsaturated fatty acids, 71% of which are due to the presence of ricinoleic acid in this oil.

The content of linoleic acid (C18:2) was significant (*p* < 0.05) in cheeses with faveleira (3.48%) and sesame oils (3.35%), indicating a good production of biohydrogenation intermediates [6]. Increased levels of linolenic acid (C18:3) in dairy products are not usual because these levels require that fatty acids coming from the diet are protected from ruminal biohydrogenation [24]. In the present study, there was a decrease (*p* < 0.05) in C18:3 n3 in the three treatments with vegetable oils compared to the control. Polyunsaturated fatty acids present in the rumen suffer less biohydrogenation because goats have a greater salivary secretion rate and consequently greater capacity to protect polyunsaturated fatty acids due to the higher rumen pH stability as a function of the buffering characteristic of saliva [5,11].

### 2.2. CLA Isomers in the Fat of Cheeses

Table 2 shows an increase (*p* < 0.05) in the levels of CLA isomers in the fat of cheeses, particularly due to the increase in C18:2 *cis-*9,*trans-*11, which almost doubled its value in treatments with faveleira (1.07) and sesame oils (0.89) compared to the control cheese (0.56) and the cheese from goats fed castor oil (0.55). The other CLA isomers were found only in trace amounts; thus, it can be inferred that with a regular diet, this isomer is poorly produced in the rumen of goats [25].

Cheeses in this study may have had an increased percentage of *cis-*9,*trans-*11-CLA due to the supplementation of animal diets with vegetable oils rich in C18:2, such as faveleira and sesame oils, these cheeses were not matured, only stored for seven days, which possibly did not contribute to this increase.

The changes observed in the *cis-*9,*trans-*11-CLA content are in agreement with the results of Santos *et al.* [12], who studied caprine Coalho cheese enriched with CLA by dietary supplementation of goats with soybean oil and observed a percentage of CLA that was 3 times higher than that in the control cheese. This isomer represents up to 90% of the total CLA of milk fat, and the results confirmed some preliminary studies carried out by Bouattour *et al.* [26] and Bernard *et al.* [5], in which the inclusion of vegetable oils in the diet of goats provided an increase over 100% (w/w) in the CLA levels in the goat milk. This information reinforces a rational approach to improve the CLA levels in milk fat with increased dietary intake of unsaturated fatty acids, which are substrates for the synthesis of ruminal CLA [14,27]. Beside, Luna *et al.* [15] emphasizes that the CLA content of processed cheese did not modify the isomer profile in these dairy products, thereby confirming the stability of rumenic acid during manufacturing.

**Table 2:** Average (area% ^1^) and relationships between saturated (SFA), monounsaturated (MUFA), polyunsaturated (PUFA) fatty acids and CLA of cheeses made with milk from goats fed with different vegetable oils.

Fatty acids (%)	Treatments (Cheese) ^2^				SEM	Significance
	Control Without oil	“Faveleira” oil	Sesame oil	Castor oil		
CLA ^3^–C18:2 *cis-9*, *trans-11trans-11*	0.56 ^b^	1.07 ^a^	0.89 ^a^	0.55 ^b^	0.055	*
CLA–C18:2 *cis-9*, *cis-*11	0.01 ^c^	0.03 ^a^	0.02 ^ab^	0.01 ^bc^	0.002	*
CLA–C18:2 *trans-*9, *trans-*11	0.01 ^b^	0.01 ^b^	0.01 ^b^	0.06 ^a^	0.005	*
CLA–C18:2 *trans-*9, *cis-*11	0.01 ^b^	0.01 ^b^	0.01 ^b^	0.12 ^a^	0.016	*
SCFA short chain fatty acids ^4^	3.19	2.58	3.07	3.00	0.139	ns
MCFA medium chain fatty ^4^ acids ^4^	25.55 ^a^	19.44 ^b^	20.05 ^b^	23.42 ^ab^	0.670	*
LCFA long chain fatty ^4^ acids ^4^	71.26 ^b^	77.98 ^a^	76.88 ^a^	73.57 ^ab^	0.725	*
SFA saturated fatty acids	69.46	64.27	64.93	66.40	0.801	ns
MUFA monounsaturated fatty acids	26.11	30.20	29.87	28.35	0.813	ns
PUFA polyunsaturated fatty acids	4.43 ^b^	5.53 ^a^	5.21 ^a^	5.19 ^a^	0.115	*
PUFA:SFA	0.06 ^c^	0.09 ^a^	0.08 ^ab^	0.08 ^b^	0.002	*
MUFA:SFA	0.38	0.47	0.46	0.44	0.016	ns
DFA ^5^	42.43 ^c^	50.86 ^a^	51.51 ^a^	45.16 ^b^	1.169	*
IA ^6^	2.55	1.76	1.85	2.33	0.125	ns
(C18:0+C18:1)/C16:0	1.31 ^b^	1.67 ^ab^	1.87 ^a^	1.21 ^b^	0.073	*

^1^ Identified by comparing the retention times of methyl esters of samples with those of standards and were quantified by normalising the areas of methyl esters and expressed as a percentage of area (%); ^2^ 0% oil (control); 4.0% “Faveleira” oil; 4.0% Sesame oil; 4.0% Castor oil; ^3^ CLA = Conjugated linoleic acid; ^4^ SCFA = (C4:0-C:9:0); MCFA (C10:0-C15:1); LCFA (C16:0-C24:0). Prandini *et al*. [8]; ^5^ Desirable Fatty Acids = MUFA+PUFA+C18:0. ^6^ Atherogenicity index = [(C12:0 + (4 × C14:0) + C16:0)]/MUFA + PUFA. SEM = standard error of the mean. * *p* < 0.05; ns—non significant. ^a,b,c^ Means followed by different lowercase letters in the same row indicate significant differences with Tukey’s test at the 5% significance level.

Despite these changes observed in the fatty acid profile, Medeiros *et al.* [28] emphasize that in general, the addition of different oils (faveleira, sesame or castor oils) to the diets of dairy goats did not promote changes in the sensory quality of the cheese produced and can be used as a dietary supplement.

### 2.3. Relationships between Fatty Acids

The addition of vegetable oils had no effect (*p* ≥ 0.05) on the percentage of short-chain fatty acids (C4 to C9), which could lead to changes in the sensory quality of cheese, because these fatty acids are responsible for relevant aromatic changes in milk and thus in cheese [29]. When pronounced, the “goat” flavour is one of the factors for refusal of dairy products; however, substances responsible for goat flavour are not well known [2]. Silanikove *et al.* [30] reported that the characteristics of goat milk flavour can be attributed to the presence of lipids in the form of short-chain fatty acids, mainly caproic (C6:0) and caprylic acids (C8:0).

Significant differences (*p* < 0.05) were observed in the decrease in total medium-chain fatty acids (MCFA), which represents the sum of C10:0 and C15:1 [8] in cheeses from goats supplemented with faveleira or sesame oil compared to control cheese. This change indicates that the addition of dietary lipids, which are derived from *de novo* synthesis, interfered with ruminal activity [16]. These results corroborate those by Santos *et al.* [31], who supplemented the diet of lactating cows with soybean oil and whole milled grain and found a reduction in medium-chain fatty acids, especially with the addition of oil in the free form, as used in this experiment. The synthesis of these fatty acids occurs in the mammary gland, with the participation of the enzymes acetyl CoA carboxylase and fatty acid synthetase, which use acetate as the main lipogenic precursor in ruminants [32]. Fernandes *et al.* [3] studied the milk profile of Moxotó crossbred goats fed diets supplemented with sunflower and cotton oils and reported that adding oil to the diet decreased the amount of medium-chain fatty acids, such as myristic acid (C14:0).

A significant increase (*p* < 0.05) was observed in the levels of polyunsaturated fatty acids (PUFA) and long-chain fatty acids (LCFA) in cheeses made with milk from goats fed with faveleira, sesame or castor oil. The increase in the fatty acids C18:0, C18:1 n9*cis*, C18:1 n9*trans*, and C18:2 n6*cis* after treatments with sesame and faveleira oils contributed to the increase in the LCFA percentage, which was 78% for faveleira oil and 77% for sesame oil compared to control cheese with only 71% of the total long-chain fatty acids. This result may be due to the biohydrogenation process that occurs from the rumen up to the mammary gland of goats [7]. For cheese made from milk from goats supplemented with castor oil, this increase in the LCFA profile was lower compared to other oil treatments due to the lower percentage of stearic and oleic acids; the PUFA percentage remained similar due to the considerable presence of 6.5% of ricinoleic acid (*p* < 0.05).

The fatty acid profile of the diet is the main factor of nutritional importance that modifies the content of long-chain fatty acids in milk [14,23]. Authors have shown that medium- and long-term supplementation with lipid sources rich in C18:2 [33] and C18:3 [13] modified the fatty acid profile of goat milk. Pereira *et al.* [6] studied the fatty acid profile of milk from goats supplemented with castor and licuri oils and confirmed the increase in long-chain fatty acids seen with this experiment. Additionally, these authors reported that the inclusion of castor oil has the advantage of increasing polyunsaturated fatty acids, which are beneficial to human health; however, these fatty acids also accentuate the rancid flavour characteristic of goat milk.

The use of vegetable oils at 4% in the diet of goats resulted in an increase (*p* < 0.05) in the DFA level (Table 2), especially due to the addition of sesame (51.51%) or faveleira oil (50.86%), followed by treatment with castor oil (45.16%), compared to control cheese (42.43%). The results for the AI were not significant (*p* ≥ 0.05) between the cheeses analyzed, while the relationship (C18:0 + C18:1/C16:0) effect was observed (*p* < 0.05), showing higher values for the treatments with cheese faveleira and sesame. The intake of monounsaturated fatty acids provides benefits for human health by causing a decrease in total cholesterol levels in blood plasma [34]; for this reason, the presence of these fatty acids is desirable in the diet, represented here with a better percentage in the cheeses from the milk of goats supplemented with sesame or faveleira oil.

## 3. Experimental

### 3.1. Cheese Samples and Production Technology

The cheese was made from the milk of crossbred Saanen × French Alpine goats, which were hand milked, kept in confinement and fed diets prepared according to NRC [35] requirements (Table 3). 

**Table 3:** Percentage and chemical composition of experimental diets.

Components	Experimental diets			
	Control Without oil	“Faveleira” Oil	Sesame Oil	Castor oil
	Proportion of ingredients (% Dry matter)			
Tifton hay	49.30	49.06	49.06	49.06
Ground corn	37.29	32.99	32.99	32.99
Soybean meal	10.79	11.34	11.34	11.34
Faveleira oil	0.00	4.00	0.00	0.00
Sesame oil	0.00	0.00	4.00	0.00
Castor oil	0.00	0.00	0.00	4.00
Core mineral ^1^	1.37	1.47	1.47	1.47
Limestone	1.25	1.13	1.13	1.13
	Chemical composition (%)			
Dry matter ^2^	86.99	87.42	87.42	87.42
Organic matter	91.91	91.96	91.96	91.96
Mineral matter	8.09	8.04	8.04	8.04
Crude protein	13.82	13.69	13.69	13.69
Ether extract	3.15	6.97	6.97	6.97
Neutral detergent fiber ^3^	39.92	39.19	39.19	39.19
Acid detergent fiber ^3^	18.70	18.48	18.48	18.48
Lignin	3.12	3.03	3.03	3.03
Total carbohydrates	74.95	71.31	71.31	71.31
Total digestible nutrients	61.61	69.68	69.05	69.19
Metabolizable energy (Mcal/kg) ^4^	2.36	2.64	2.67	2.60

^1^ Mineral supplement (nutrients/kg de supplement): Ca = 210 g; P = 70 g; Mg = 5 g; F = 700.00 mg; Zn = 3.010 mg; Cu = 440 mg; Mn = 1,485 mg; Co = 25 mg; Fe = 340 mg; Cr = 6.00 mg; Se = 20 mg; I = 48 mg; S = 10 g; Vit. A = 250,000.00 UI/kg; Vit. D3 = 40,000.00 UI/kg; Vit E = 350.00 UI/kg. ^2^ % on the basis dry matter; ^3^ Free ash and protein; ^4^ EM (Mcal/kg) **=** ED (Mcal/kg) × 0.86.

There were four diets used in the study: 1—basal diet without the addition of vegetable oil to the dry matter, 2—basal diet plus 4% faveleira oil, 3—basal diet plus 4% sesame oil, and 4—basal diet plus 4% castor oil. The seeds of faveleira stand out due to the presence of unsaturated fatty acids, especially linoleic acid (C18:2 n6 *cis*), which accounts for 63.8% of the linoleic acid. Castor seeds show 71.8% ricinoleic acid (C18:1 *cis*-9,12-OH) and sesame seeds produce oil rich in unsaturated fatty acids (48.1% of linoleic acid and 36.5% of oleic acid) [4].

The trial lasted 76 days and followed a Latin square design (4 × 4), with four treatments and four processing periods. In each period, the cheeses were made using 10 L of goat milk per treatment. The milk samples were pasteurised at 65 °C (±1 °C) for 30 min followed by cooling to 37 °C (±2 °C) and coagulation with the following additives in this sequence: 0.25 mL/L 85% lactic acid solution, 0.1 g/L lyophilised lactic starter culture with *Lactococcus lactis* subsp. *cremoris* and *Lactococcus lactis* subsp. *lactis* (R-704, Christian Hansen Ind. & Com. Ltd., Valinhos, SP, Brazil), 0.5 mL/L 50% calcium chloride, and 0.9 mL/L commercial coagulant (Ha-La^®^, Christian Hansen Ind. & Com. Ltd.). After 40 min of rest, the curd was gently cut into cubes, drained, and salted in brine (9 g/L NaCl). Then, the cheese mass was distributed into 250 g perforated moulds, pressed for 4–6 h at room temperature, vacuum packed and stored under refrigeration 4 ± 1 °C for 7 days. After this period, the fatty acid profile was analysed.

### 3.2. Analysis of the Fatty Acids Profile of Goat Cheese

The determination of the fatty acids profile in the goat cheese started with the extraction of total fat, according to the procedure described by Folch, Lees and Stanley [36], followed by saponification and esterification steps [37].

The fatty acid profile of esterified cheese samples was determined in a gas chromatograph Varian 430-GC with a flame ionisation detector (FID) and fused silica capillary column (CP WAX 52 CB Varian), with dimensions of 60 m × 0.25 mm × 0.25 mm film thickness. Helium was used as the carrier gas (flow rate of 1 mL/min). The oven temperature started at 100 °C and was programmed to increase by 2.5 °C/min to reach a final temperature of 240 °C and then maintain that temperature for 20 min for a total of 76 min. The injector temperature was maintained at 250 °C and the detector temperature at 260 °C. An aliquot of 1.0 µL of the esterified extract was injected into a split/splitless injector.

The chromatograms were recorded on Galaxie Chromatography Data System software. Fatty acids were identified by comparing the retention times of methyl esters of samples with those of standards from a Supelco ME19-Kit (Fatty Acid methyl Esters C6-C24) and with CLA (*cis*/*trans* linoleic acid methyl ester mix). The results were quantified by normalising the areas of methyl esters and expressed as a percentage of area (%).

The relationships PUFA/SFA, MUFA/SFA, (C18:0 + C18:1)/C16:0, the atherogenicity index (AI) were calculated according to Ulbright and Southgate [38], and the percentage of desirable fatty acids (DFA), expressed as the sum of unsaturated fatty acids plus stearic acid [39]. 

### 3.3. Statistical Analysis

A simultaneous double Latin square (4 × 4) experimental design was used, with 8 animals randomly distributed into 4 treatments and 4 periods. The statistical model used in the analysis of the fatty acid data was the following:

*Yijkl* = *μ* + *Qi* + *Tj* + *Pk* + *A(i)l* + *QTij* + *ξijk*

where *Yijkl* is the observation of goat *j* in period *k* submitted to treatment *i*, with *i, j,* and *k* = 1, 2, 3, or 4; *μ* is the general effect of the mean; *Qi* is the effect of the Latin square (*Q* = 1,2); *Ti* is the effect of treatment *i*, with *i* = 1, 2, 3, or 4; *Pk* is the effect of period *k; A* is the effect of goat *l* in square *i*, with *l* = 1, 2, 3, or 4; *QT* is the interaction of the effect of Latin square *i* with treatment *j*; and *ξijk* is the random error associated with each observation *Yijkl*. Treatment means were compared by Tukey’s test at 5% error probability using statistical software [40].

## 4. Conclusions

Lipid supplementation with faveleira, sesame or castor oil in the diet of dairy goats did not change the total fat content of cheeses; however, supplementation promoted a decrease in the percentage of saturated fatty acids C12:0, C14:0 and C16:0 and an increase in the percentage of PUFA and LCFA. The use of sesame and faveleira oils caused an increase in the DFA, oleic acids, linoleic acid and *cis-*9, *trans-*11-CLA profiles in goat cheese, indicating that utilization the diet of these goats can be a way of controlling the physicochemical characteristics of milk and consequently its derivatives, such as cheese.
